# Enhancing multiclass brain tumor diagnosis using SVM and innovative feature extraction techniques

**DOI:** 10.1038/s41598-024-77243-7

**Published:** 2024-10-29

**Authors:** Mustafa Basthikodi, M. Chaithrashree, B. M. Ahamed Shafeeq, Ananth Prabhu Gurpur

**Affiliations:** 1 Department of Computer Science & Engineering, Sahyadri College of Engineering & Management, Mangaluru, India; 2 Department of Computer Science and Engineering, Manipal Institute of Technology, Manipal Academy of Higher Education, Manipal, 576104 Karnataka India

**Keywords:** Multiclass, Feature extraction, SVM, LBP, HOG, PCA, MRI image, Computational biology and bioinformatics, Mathematics and computing

## Abstract

In the field of medical imaging, accurately classifying brain tumors remains a significant challenge because of the visual similarities among different tumor types. This research addresses the challenge of multiclass categorization by employing Support Vector Machine (SVM) as the core classification algorithm and analyzing its performance in conjunction with feature extraction techniques such as Histogram of Oriented Gradients (HOG) and Local Binary Pattern (LBP), as well as the dimensionality reduction technique, Principal Component Analysis (PCA). The study utilizes a dataset sourced from Kaggle, comprising MRI images classified into four classes, with images captured from various anatomical planes. Initially, the SVM model alone attained an accuracy(acc_val) of 86.57% on unseen test data, establishing a baseline for performance. To enhance this, PCA was incorporated for dimensionality reduction, which improved the acc_val to 94.20%, demonstrating the effectiveness of reducing feature dimensionality in mitigating overfitting and enhancing model generalization. Further performance gains were realized by applying feature extraction techniques—HOG and LBP—in conjunction with SVM, resulting in an acc_val of 95.95%. The most substantial improvement was observed when combining SVM with both HOG, LBP, and PCA, achieving an impressive acc_val of 96.03%, along with an F1 score(F1_val) of 96.00%, precision(prec_val) of 96.02%, and recall(rec_val) of 96.03%. This approach will not only improves categorization performance but also improves efficacy of computation, making it a robust and effective method for multiclass brain tumor prediction.

## Introduction

The Central Nervous System manages various bodily functions^1^. The brain is vital for decision-making, which makes brain tumors a serious threat to life^2^. A tumor in the brain is characterized by the incorrect growth or accumulation of cells inside the brain. To how much extent these tumors might affect the life a person is depend on factors like size of that tumor, type of that tumor and also location of the tumor and the accessibility of effective treatment options^3^. These can develop in various areas of the brain and frequently remain without symptoms until they reach more advanced stages^4^. Pinpointing the correct position of a tumor can be quite challenging for radiologists. Typically, CT or MRI scans are utilized to detect and visualize the tumor within the brain. Before brain surgery, a biopsy is often conducted as an initial clinical procedure to extract brain cells for examination. Precise measurement and diagnosis of a brain tumor are critical to avoid any technical or qualitative errors^5^. Quick prediction of these kind of tumors is very much necessary to enhance the medical outcomes. MRI is the most widely used method for diagnosing these tumors due to its detailed imaging capabilities. The use of contrast-enhanced MRI is particularly effective, as it provides more detailed information about the tumor, leading to more accurate diagnoses. Current research is focusing on further enhancing MRI diagnostics by incorporating contrast agents. Additionally, Computed Tomography (CT) scans are valuable for revealing the internal structure of organs, including the brain. However, the manual segmentation of tumors, which requires the expertise of radiologists, is a very difficult process, especially given the vast amount of MRI data. These challenges highlight the need for automatic brain tumor segmentation, which aims to reduce errors and streamline the diagnostic process^6^.

Gliomas are one type of brain tumor. Pituitary adenomas and meningiomas form the remaining types of brain tumors^7^. Meningiomas start near the brain’s outer lining, where as gliomas form surrounding glial cells, and pituitary tumors develop within the pituitary gland. Effective treatment depends on getting the diagnosis right^1^. The tremendous increase in deaths caused by brain tumors in the past few years shows how important it is to diagnose them correctly. Since performing biopsies on brain tumors is challenging, MRI is commonly used for detecting such conditions^8^. MRI has gained widespread recognition as the primary noninvasive technique for detecting brain tumors, because its exceptional capability to differentiate soft tissues^9^. According to the Brain Tumor Association of America there are 24,530 new cases occurring every year. However, these numbers are estimates and may not reflect the exact numbers of brain tumor cases in the United States^10^.For the diagnosis of this lethal disease, the computer vision imaging techniques are most commonly used^11^. The development of these technologies greatly impacts the medical field^12^. Image classification is one of the machine learning (ML) method which used to instruct the system. It uses classified medical images for diagnosis and training purposes^13^. Deep learning (DL) is the newly developed technique having more popularity in every field^14^. The advancements in both of these techniques offer better solutions for the diagnosis of brain tumors.

Numerous research efforts are underway to develop models for accurately predicting brain tumors. While most prior research has primarily focused on classifying brain tumors into two categories, we have extended the classification to four categories. This task becomes more difficult because certain tumor types exhibit greater similarities.

To make brain tumor classification more precise, we use the advanced Support Vector Machine (SVM) algorithm along with the techniques used for extraction of features and reduction of dimensions. Extracting features is crucial for identifying key details from medical images. In this research, we use HOG and LBP as techniques to extract features, helping the computer identify subtle differences and improving the performance of the SVM model. Additionally, we use Principal PCA to minimize the size of data.

Although our findings show only a slight difference in accuracy between the model with PCA and the one without, including PCA makes data processing more efficient.

## Literature review

A brain tumor poses risk to human life if it is not detected early. As technology advances, various methods have been used to improvise the efficiency of detection of this lethal disease.

Hashemzehi et al^15^. devised a novel technique merging different neural networks to identify brain tumors. They made use of MRI images. They achieved a remarkable accuracy of 95% in classifying various types of brain tumors.

Saxena et al^16^. focused on binary classification to distinguish between two categories of brain tumors. Using pre-trained neural network models, they achieved an accuracy of 95%. Additionally, their study explored parameter adjustments to enhance model accuracy. Musallam et al^17^. addressed the challenge of classifying multiple types of brain tumors by introducing a novel neural network architecture named Deep CNN. Their model demonstrated exceptional accuracy in tumor classification. Gomez-Guzman et al^18^. extended previous research by evaluating the performance of seven neural network models. Their findings highlighted the promising results of the InceptionV3 model in properly identifying brain tumors, suggesting its potential clinical utility for early tumor detection. Wadhah et al^19^. presented a brain tumor classification system integrating various techniques to enhance MRI image quality and extract discriminative features. Utilizing SVM for classification, they got an acc_val of 90.27%. Amin et al^20^. addressed the challenge of early detection of brain tumor by employing SVM for classification. Through rigorous data preprocessing, they got an average acc_val of 97.1%. Tazin et al^21^.focused on utilizing pre-trained models for identification of brain tumor. They achieved a high accuracy of 92% with the MobileNetV2 model by optimizing training parameters, emphasizing the importance of meticulous strategy implementation. Glory Precious et al^22^. introduced a new neural network model, Lightweight Sequential net, and compared its performance with established models. Their results demonstrated promising accuracies ranging from 79.54 to 91.73%, indicating potential for accurate brain tumor identification. And Nayak et al^23^. continued the exploration of CNNs with a dense EfficientNet, outperforming other models with high testing accuracy. Their focus on efficiency and parameter minimization addressed computational challenges in brain tumor classification. Ramaha et al^24^. brought together machine learning techniques, combining deep CNN with transfer learning and SVM for two class classification. With notable accuracies of 97% for SVM and 98% for CNN, this study recommended ensemble classification approaches for further refinement. Waghmere et al^25^. utilized the VGG16 model for brain tumor detection. Prior to CNN input, the images underwent preprocessing and augmentation. The system attained an accuracy of 95.71%. Sohaib Asif et al^26^. extended the research to multiclass categorization, achieving remarkable accuracies of 99.67% on three-class dataset and 95.87% on the four-class datasets. Their Xception-based model set a benchmark for future experiments. In the context of multiclass classification, Mrinmoy Mondal et al^27^. focused on refining diagnostic methodologies by using VGG-19 with transfer learning. The proposed framework achieved an acc_val and F1_val of 94%.

Addressing category-based classification, S Deepak and P M Ameer et al^28^. combined CNN features with SVM, yielding an impressive overall classification accuracy of 95.82%. Their work not only contributed to advancements in multiclass brain tumor classification but also emphasized the computational advantages of the CNN-SVM approach. Yazdan et al^29^. utilized multiscale CNN on MRI images, categorized into meningioma, pituitary, glioma, and no tumor classes, achieving an accuracy of 91.2%. On the same dataset, Mahjoubi et al^30^. applied CNN, achieving an accuracy of 95.44%. Their future work includes creating more perfect models and making use of larger and also diverse datasets. Saeedi et al^31^. utilized an openly available dataset containing T1-weighted contrast-enhanced MRI images, categorized into meningioma, glioma, pituitary, and healthy classes. They applied 2D CNN and got an acc_val of 93.44%. Future work includes exploring the development of efficient neural networks for quick and accurate identification, integrating diverse algorithms.

While existing studies as described above and detailed in Table 1 have made significant strides in brain tumor classification, they often fall short in addressing the challenges of multiclass classification and computational efficiency. Many approaches focus on binary or ternary classification, which, although valuable, do not fully capture the complexity of distinguishing between multiple tumor types. Moreover, a significant portion of these studies rely on deep learning techniques, which, despite their accuracy, require substantial computational resources and extended training times. This reliance on resource-intensive methods can be prohibitive in real-world clinical settings, particularly where access to high-performance computing is limited.

Our research bridges these gaps by introducing a multiclass classification framework that is both accurate and efficient. By using SVM along with efficient feature extraction methods, our approach reduces computational overhead while maintaining high classification accuracy. This makes our model well-suited for implementation in resource scarce environments. Additionally, by expanding the classification to multiple tumor types, our model offers a more comprehensive and clinically relevant diagnostic tool, addressing the limitations of existing studies and aligning with the nuanced needs of medical practice.

**Table 1:** Comparison of performance of reviewed papers.

Ref	Year	model	Dataset used and number of classes	Future Work
^15^	2020	CNN-NADE	Real world dataset having 3 classes	In future research, there is potential for integrating preprocessing methods and PCA with CNNs for classification purposes
^19^	2022	SVM	Real world dataset having 3 classes	Future research will explore the use of data collected from various imaging modalities, with a focus on validating its effectiveness
^20^	2020	SVM	Harvard dataset, RIDER brain image data, real world dataset having 2 classes	Incorporating additional imaging modalities and extending the evaluation to encompass more classes is part of the future research
^21^	2021	MobilenetV2	Kaggle-brain tumor dataset having 2 classes	Future work will center on expanding the study to larger datasets and integrating more pretrained models to enhance performance
^28^	2021	CNN-SVM	Figshare open dataset having 3 classes	In the future, the focus will be on adapting the system for image retrieval, enhancing performance through extensive data augmentation, and fine-tuning the classifier for real image testing to validate its diagnostic applicability
^26^	2023	Xception	Kaggle- MRI Dataset having 4 classes	Future work includes experimenting with additional brain MRI data to expand the dataset while maintaining performance levels
^29^	2022	Multiscale CNN	Kaggle-MRI Dataset having 4 classes	In future research, the focus will be on optimizing the deep learning model for noise robustness, exploring alternative architectures, and validating its performance across diverse datasets and clinical settings
^30^	2023	CNN	Kaggle-MRI Dataset having 4 classes	Enhancing the robustness and accuracy of models will involve making use of alternative methods and utilizing larger datasets
^31^	2023	2D CNN	Openly available dataset having 4 classes	Future work involves combining different models to improve the performance

## Methodology

**Fig. 1:**
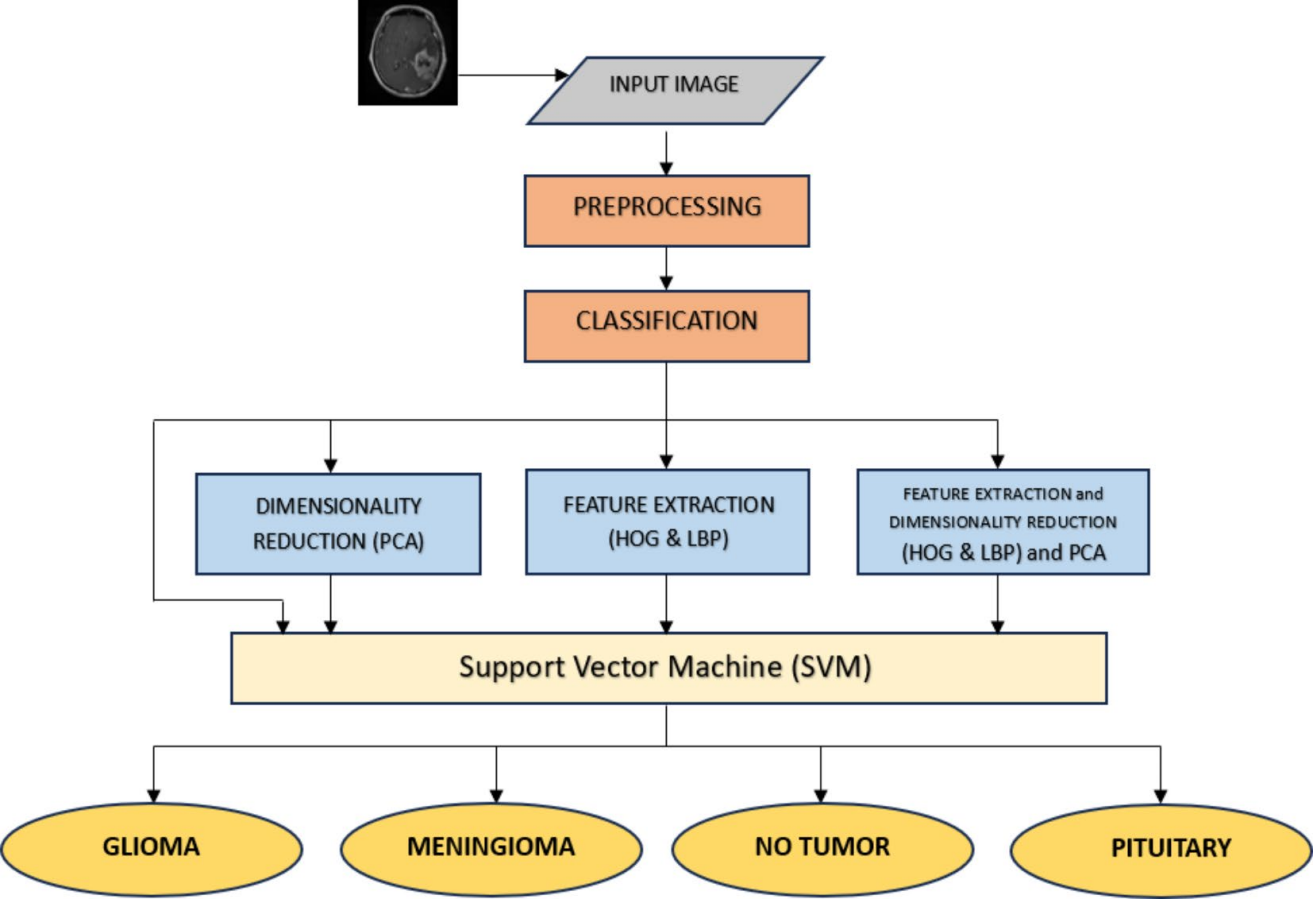
Schematic diagram illustrating the categorization process.

As seen in Fig. 1, the methodology involves the classification of four distinct classes. The initial phase employs SVM as the primary classifier. Subsequently, the process unfolds with the integration of SVM and PCA for dimensionality reduction. Following this, HOG and LBP are applied for feature extraction. Finally, a combined approach utilizing HOG, LBP, and PCA is implemented.

### Dataset

**Fig. 2:**
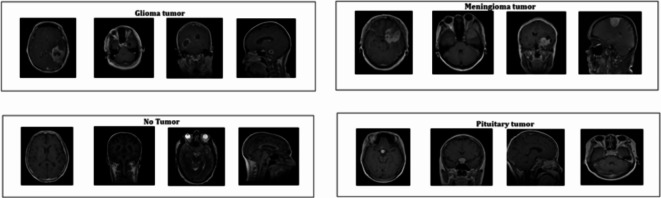
Sample MRI Images of Various Planes from the Dataset.

Dataset is obtained from Kaggle which is a combination of three different datasets. This dataset has 7023 images of brain MRI in total which are classified into training and testing folders as shown in Table 2. There are 4 classes namely glioma(Class G), meningioma(Class M), no tumor(Class N), and pituitary(Class P) in both the folders. As seen in Fig. 2, it includes MRI images taken from various planes. Table shows the class wise distribution of images in dataset.

**Table 2:** Class-wise distribution of images in dataset.

Classes	Training	Testing
Class G	1321	300
Class M	1339	306
Class N	1595	405
Class P	1457	300

### Models and techniques

SVM is the main model used, and it is accompanied by techniques such as HOG, LBP for extracting the feature, and PCA for reducing the dimensionality.

#### Support vector machine (SVM)

The SVM algorithm is employed for categorization tasks, generating decision boundaries known as hyperplanes to segregate datasets. SVM is versatile in handling data containing both linear and nonlinear relationships. In instances where linear separation is feasible, the dataset is partitioned into two distinct groups by the hyperplane. However, when linear separation isn’t feasible, SVM employs a technique known as the kernel trick. Through this method, SVM transforms initial input space into a feature space of higher dimensionality where effective separation of data may be achieved^7^.

#### Histogram of oriented gradients (HOG)

Lee and Chung proposed HOG characteristics, inspired by the concept of object shape and state. This approach involves characterizing an object through the pixel intensity distribution and direction, referred as a gradient vector^32^. Systems designed for recognizing objects make use of HOG for image classification. In medical imaging, analyzing the frequency of different gradient orientations in a particular region helps identify patterns. The HOG feature extraction plugin simplifies the collection of these features, providing a straightforward and efficient method^24^.

HOG feature is calculated as follows.

Given an image divided into M×N cells, each cell containing m×n pixels, and orientation bins B in the histogram:

1.For each cell.

Compute the gradient magnitude (mag_x_,mag_y_) and orientation (θ) for each pixel.

Calculate the histogram of gradient orientations (HOG histogram) for the cell by accumulating gradient magnitudes into orientation bins.

2.For each block (comprising P×Q cells).

Normalize the histograms of all cells within the block, typically using L2 normalization.

Concatenate the normalized histograms from all cells within the block to form a block-level feature vector.

Concatenate the block-level feature vectors from all blocks to obtain the final HOG feature vector representing the image.

#### Local binay pattern(LBP)

Ojala et al. introduced LBP. LBP has gained widespread use in feature extraction due to its simplicity in calculation and ease of extraction. This method finds extensive application in machine vision detection^33^.

Given a central pixel P_c_ and its neighbouring pixels P_i_ for i = 0 to till N-1 were N is the sampling point numbers.

1 $$LBP(P_{C})\sum_{i=0}^{N-1}\,s(P_i-P_c)X\;2^i$$

s(x) is referred for the sign function, which returns 1 if x is greater than or equal to 0 and 0 otherwise.

P_i_-P_c_ is the intensity difference between the neighboring pixel P_i_ and central pixel P_c_ .

This formula represents the process of computing the LBP value for a single pixel in the image.

**Fig. 3:**
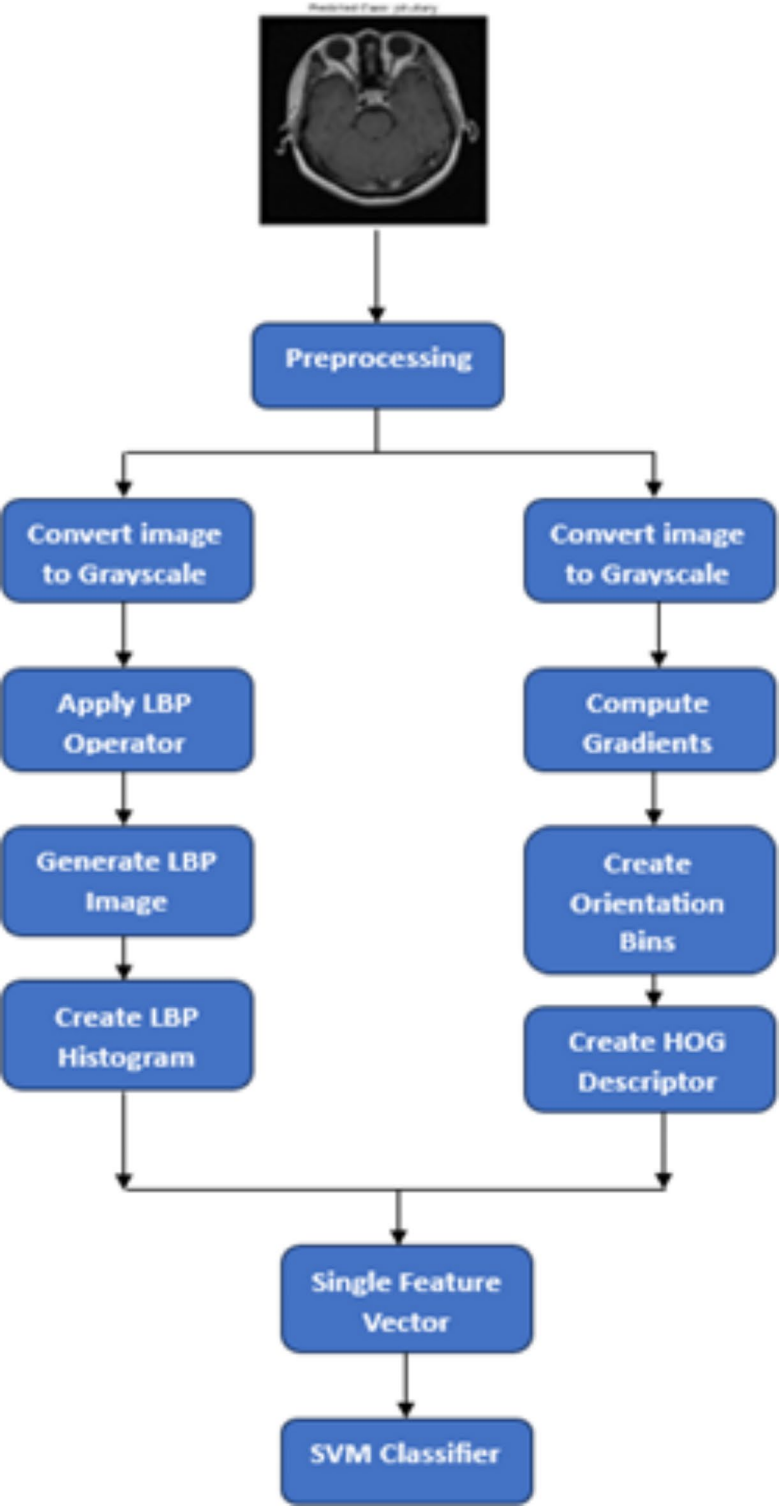
Flowchart describing the Feature Extraction Process.

Figure 3 displays the original image with lbp feature and hog feature extraction.

#### Principal component analysis (PCA)

PCA is an algorithm used for extracting the features and reducing the dimensions. This method involves a linear transformation of the features, reducing their dimensions from high to low^34^.

Formula for PCA is given by.

2 $$PC_x=\sum_{y=1}^n\,W_{xy}Z_y$$

Where PC_x_ means x-th principal component, W_xy_ means the weight of the y-th feature in the x-th principal component, and Z_y_ is the y-th original feature, n is the total number of original features.

This formula represents a linear combination of the original features weighted by the corresponding weights W_xy_ to obtain the x-th principal component.

### Coding and experimentation

In our multiclass classification, remarkable outcomes are achieved with SVM utilizing HOG, LBP, and PCA. This section provides detailed insights into the coding and experimentation processes of this high-accuracy model.

#### Preprocessing

In the preprocessing stage of the brain tumor classification methodology, each class undergoes a series of systematic processing steps to ensure consistency of input data and to improve input data’s quality. The key preprocessing steps are as follows.

##### Reading and resizing images

Grayscale medical images, representing different classes of brain tumors, are initially read from the dataset. Since the images may vary in resolution, they are resized to 64 × 64 pixels. This uniformity in image size helps streamline the feature extraction process and ensures that the classifier receives inputs of consistent dimensions.

To ensure robustness in the preprocessing pipeline, error-handling mechanisms are implemented. These mechanisms detect and manage any issues encountered during image processing, such as corrupted files or unsupported formats, preventing disruptions in the workflow.

##### Flattening images

After resizing, the images are flattened into one-dimensional arrays. Flattening converts the 2D pixel matrix into a 1D vector, which simplifies the data structure for subsequent processing stages. This transformation is particularly useful when feeding data into machine learning models that require vectorized input.

##### Data Augmentation

It is a critical preprocessing step aimed at increasing the size of the training dataset. In medical imaging, the available dataset is often limited, so augmentation techniques are used.

Images are rotated, horizontally and vertically flipped or zoomed to increase the training dataset zize allowing the model to detect tumors of varying sizes.

##### Normalization

To further enhance the preprocessing, image’s pixels are normalized. This step scales the pixel values to a specific range (0 to 1), which helps to make the training process quicker.

##### Histogram Equalization

This is applied to improvize the contrast in cases where images suffer from poor contrast, making the tumor regions more distinguishable from surrounding healthy tissue. This step adjusts the intensity distribution of the image, improving the visibility of important features.

After completing the preprocessing steps, the images are prepared for extraction of features.

#### Feature extraction

**Fig. 4:**
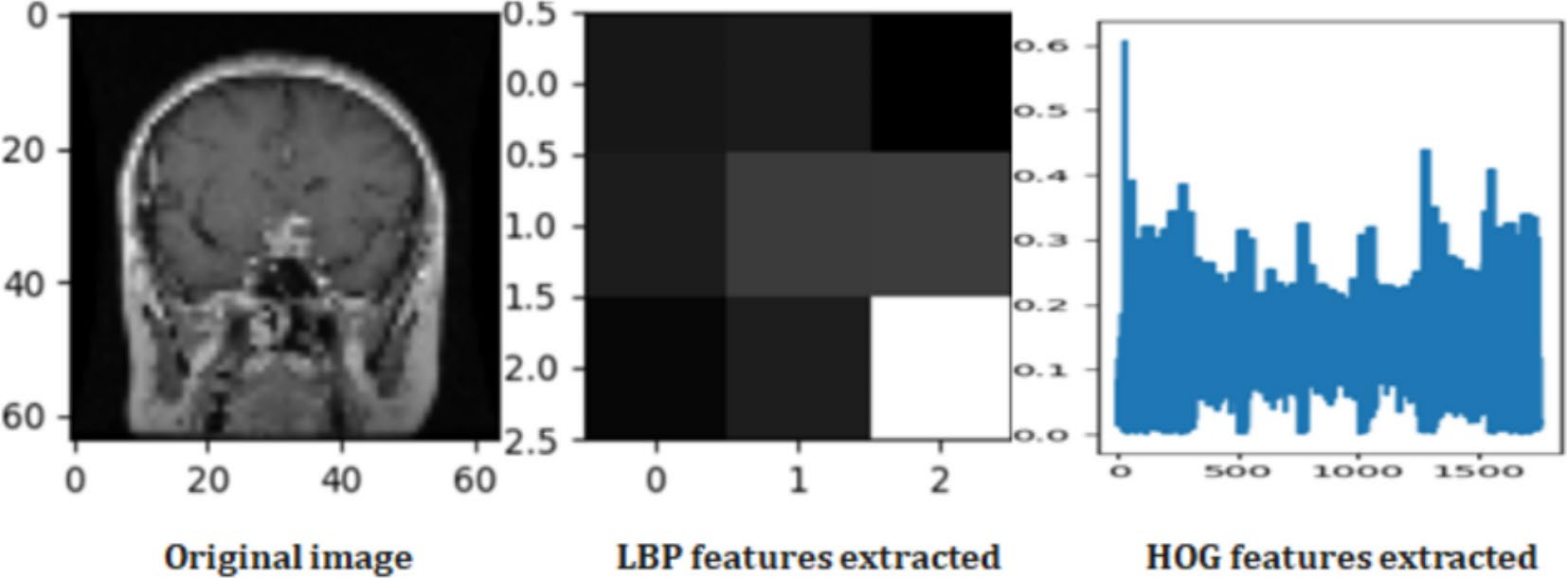
Original Image with LBP and HOG Feature Extraction.

As shown in Fig. 3, Grayscale medical image processing for brain tumor classification involves two key feature extraction methods. First, LBP captures texture patterns, forming a distinctive normalized histogram-based feature vector. Second, the HOG extracts shape information, by creating a flattened one-dimensional array. Figure 4 shows the original Image with LBP and HOG feature extraction.

##### Local binary patterns (LBP)

Is a widely used method, particularly effective for capturing texture patterns in grayscale images. LBP works by matching each of the pixels with its neighbors, generating a binary pattern based on intensity which is then altered into a decimal value, indicating the texture. The procedure is repeated across the entire image, and the frequency of each pattern is recorded in a histogram, resulting in a normalized feature vector.

The robustness of LBP to illumination changes also adds to its effectiveness in medical imaging, where consistent lighting conditions are not always guaranteed.

##### Histogram of oriented gradients (HOG)

This method is primarily used to extract shape and structural information from images. HOG first devides the image into tiny cells and then it calculates the gradient direction and magnitude of those cells which is then altered into an exact number of orientation bins, creating a histogram that describes the local shape characteristics. Then it forms a feature vector which captures the distribution of edge directions within the image.

In brain tumor classification, HOG is crucial because tumors often exhibit distinct shapes and structural features compared to surrounding healthy tissue. By focusing on the edges and contours within the image, HOG effectively captures these differences, contributing to the accurate identification and classification of tumors.

##### Combined feature representation

To create a more robust and comprehensive feature representation for brain tumor classification, the LBP and HOG feature vectors are concatenated. This unified feature vector integrates both texture and shape information, offering a more complete descriptor of the image. While LBP emphasizes the fine-grained texture details, HOG provides complementary information about the overall shape and structure.

The combination of LBP and HOG allows for the extraction of rich features that encapsulate both the micro-level texture patterns and macro-level shape characteristics of brain tumors.

#### Data preparation and dimensionality reduction

During data preparation, features and labels undergo transformations for model training. The dataset is devided into a set of training data and a set of testing data. Furthermore, standardizing feature vectors eliminates scale variations, prevents larger-magnitude features from dominating, and improves the overall stability of the model. Principal Component Analysis (PCA) reduces dimensionality, retaining 95% variance for a concise data representation. Transformed feature vectors from PCA serve as input for model training. A grid search fine-tunes the SVM, optimizing parameters for enhanced classification on the dataset. In summary, data handling and dimensionality reduction prepare for efficient brain tumor classification.

#### Model training

During the training phase, the dataset is meticulously prepared and features are extracted, resulting in standardized and dimensionality-reduced feature vectors. The dataset is then divided into a set of training data and a set of testing data, with the former teaching the SVM model to detect the patterns and their relationships within the data. This model’s parameters are optimized through grid search, exploring various combinations of regularization parameters and kernel types to maximize predictive accuracy. The fine-tuning process ensures the model generalizes well on unseen data, preventing overfitting.

#### Evaluation and validation

The evaluation phase assesses the SVM model on a separate testing dataset with unseen brain tumor images. The testing data undergo preprocessing, and features are extracted using LBP and HOG techniques. Standardized, dimensionality-reduced test data is input into the SVM model with an optimized configuration. Post-testing, acc_val, prec_val, rec_val, and F1_val are computed. Validation sets, initially used for training and monitoring, now fine-tune models for optimal generalization.

#### Pseudocode


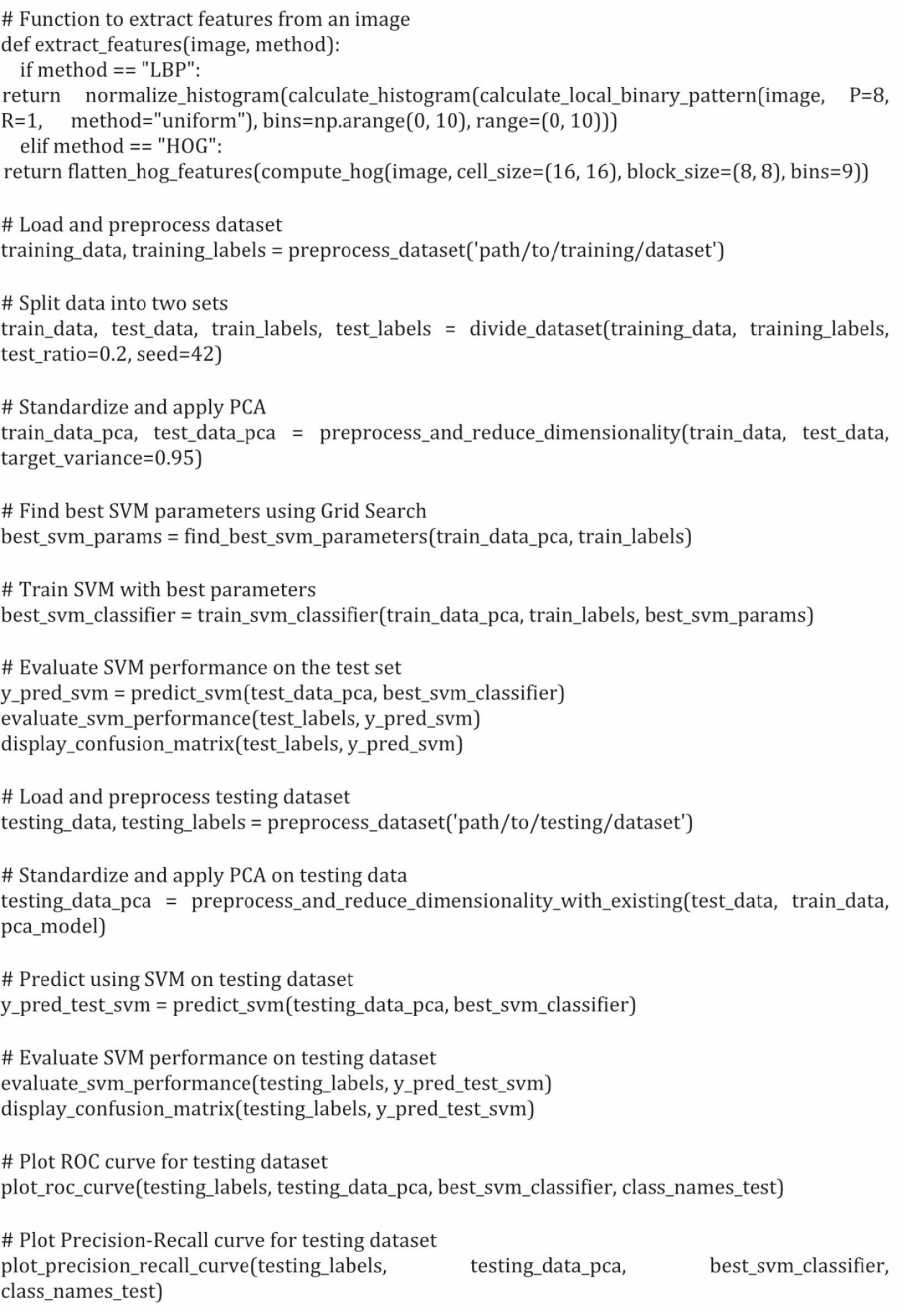


## Results and discussion

In our research, we have used the Support Vector Machine classifier and evaluated its performance using various techniques. Our project was conducted on a dataset obtained from Kaggle, having 7023 MRI images categorized into four classes. The dataset has images taken from different planes.

There are training and testing folders in the dataset as shown in Table 2.

We started the classification process by dividing the training set into 80% training and 20% validation subsets. Then efficacy of the classifier was evaluated on the test_set. The SVM classifier attained an acc_val, F1_val, prec_val, and rec_val of 86.57%, 86.29%, 86.57%, and 86.36%, respectively.

Next we have used PCA in conjunction with SVM. This approach resulted an improved performance, with an acc_val, F1_val, prec_val, and rec_val of 94.20%, 94.15%, 94.13%, and 94.20%, respectively.

The next step was applying feature extraction before classifying using SVM. LBP and HOG Feature extraction techniques were used for it. This method yielded an accuracy, F1 score, precision and recall of 95.95%, 95.93%, 95.94%, and 95.95%, respectively.

At last, we made use of PCA for dimensionality reduction along with feature extraction before classifying using SVM. This method showed an improved accuracy, F1 score, precision and recall of 96.03%, 96.00%, 96.02%, and 96.03%, respectively.

We observed only marginal difference in accuracy when we incorporated PCA but it provided other benefits such as reduced overfitting and computational efficiency. Table 3 shows the metric values of the approaches as mentioned above.

**Table 3:** Performance Metrics for SVM classifiers.

Method	Acc_val	F1_val	Prec_val	Rec_val
SVM	86.57%	86.29%	86.57%	86.36%
SVM with PCA	94.20%	94.15%	94.13%	94.20%
SVM with HOG, LBP	95.95%	95.93%	95.94%	95.95%
SVM with HOG, LBP And PCA	96.03%	96.00%	96.02%	96.03%

**Fig. 5:**
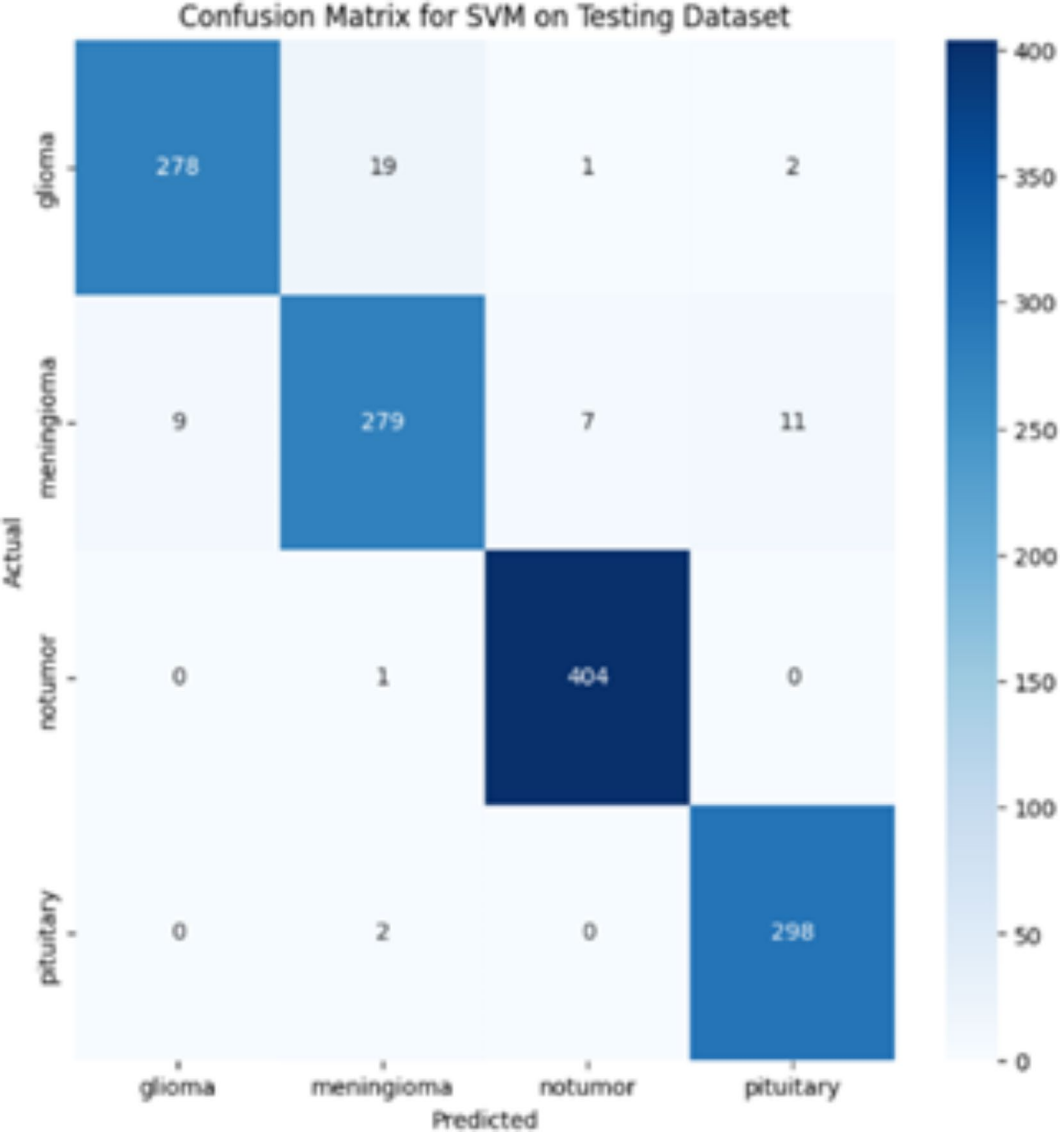
Confusion Matrix of SVM with HOG, LBP, PCA on Test Dataset.

Figure 5shows the confusion metric on unseen test data for the high accuracy model. The test dataset is having 300 glioma, 306 meningioma, 405 no tumor, and 300 pituitary images. Confusion matrix tells us how accurate is our model at identifying different types of brain tumors. Diagonal of the confusion matrix has numbers which indicates how many instances of those classes are correctly identified. According to the figure, our model could accurately identify 278 cases of glioma tumor types, 279 cases of meningioma tumor types, 404 cases of no tumor and 298 cases of pituitary tumors. Off diagonal numbers indicate misclassification. By looking at the off diagonal numbers we can make out where we need to do improvement^23^.

**Fig. 6:**
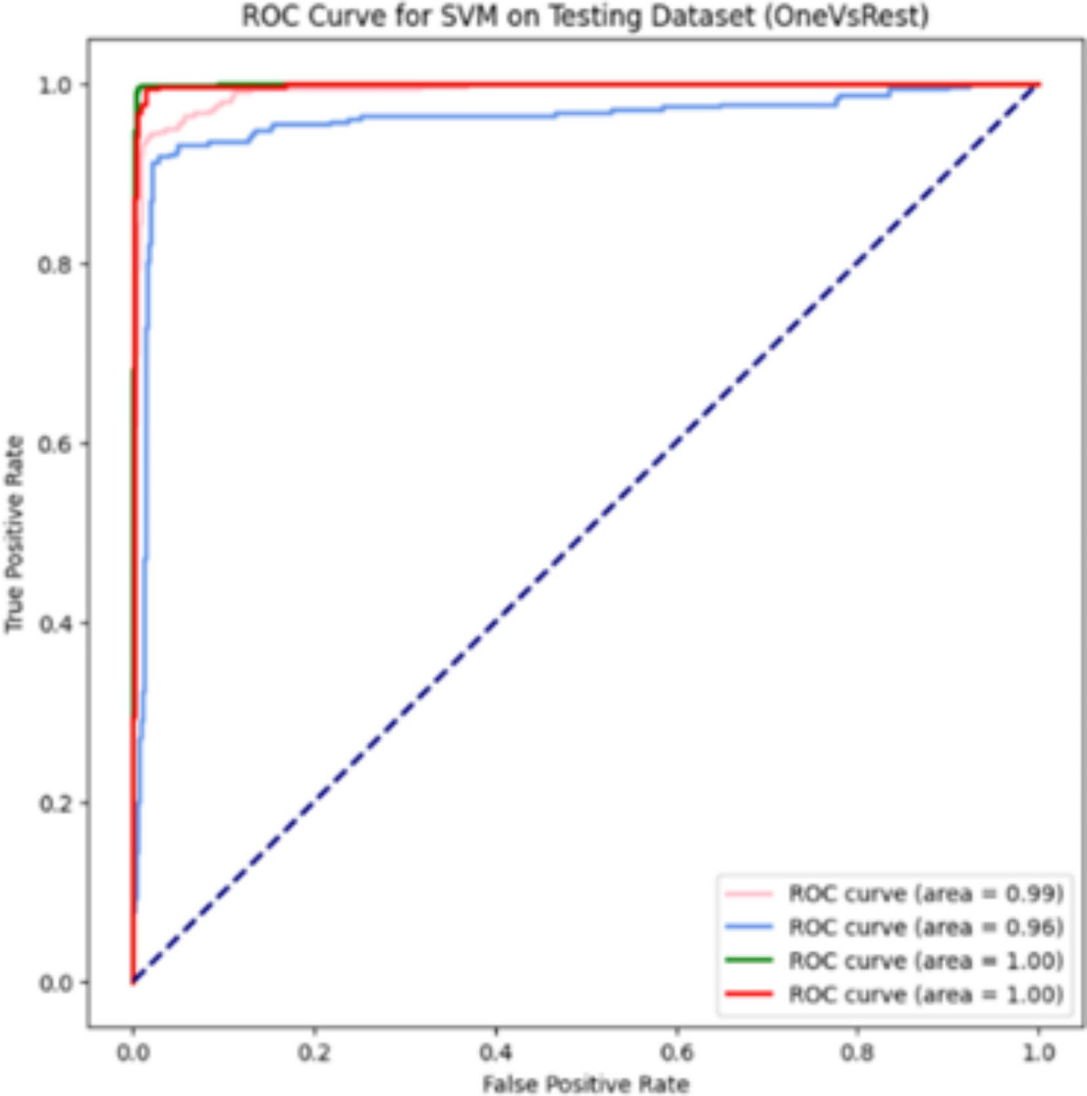
ROC Curve of SVM with HOG, LBP, PCA on Test Dataset.

Figure 6, shows the graph of ROC(Receiver operating characteristic) curve of high accuracy model. For each classification scenario, we analyze the area beneath the ROC curve also termed as AUC to quantify the model’s diagnostic accuracy. A higher AUC value suggests better model performance. ROC curve here shows how this high accuracy model identifies different types of brain tumors. There are lines to represent tumor types in the graph: Class G (pink), Class M (blue), Class N (green), and Class P(red). The ROC curve for Cass G has an AUC of 0.99, indicating that the model can correctly identify glioma cases while minimizing false positives.The AUC for meningioma is 0.96, which also represents a strong performance, though slightly lower than that for glioma. This indicates that the model is very effective at distinguishing meningioma cases, with a minor degree of misclassification compared to glioma.The ROC curve for cases with no tumor has reached an AUC of 1.00, signifying perfect classification. This means the model identifies all cases without a tumor accurately, with no false positives or false negatives.Similarly, the AUC for pituitary tumors is also 1.00, demonstrating perfect classification performance for this tumor type as well.

The ROC curves indicate that the model is particularly adept at handling complex multiclass classification tasks, with an almost flawless ability to differentiate between tumor types and normal cases^35^.

**Fig. 7:**
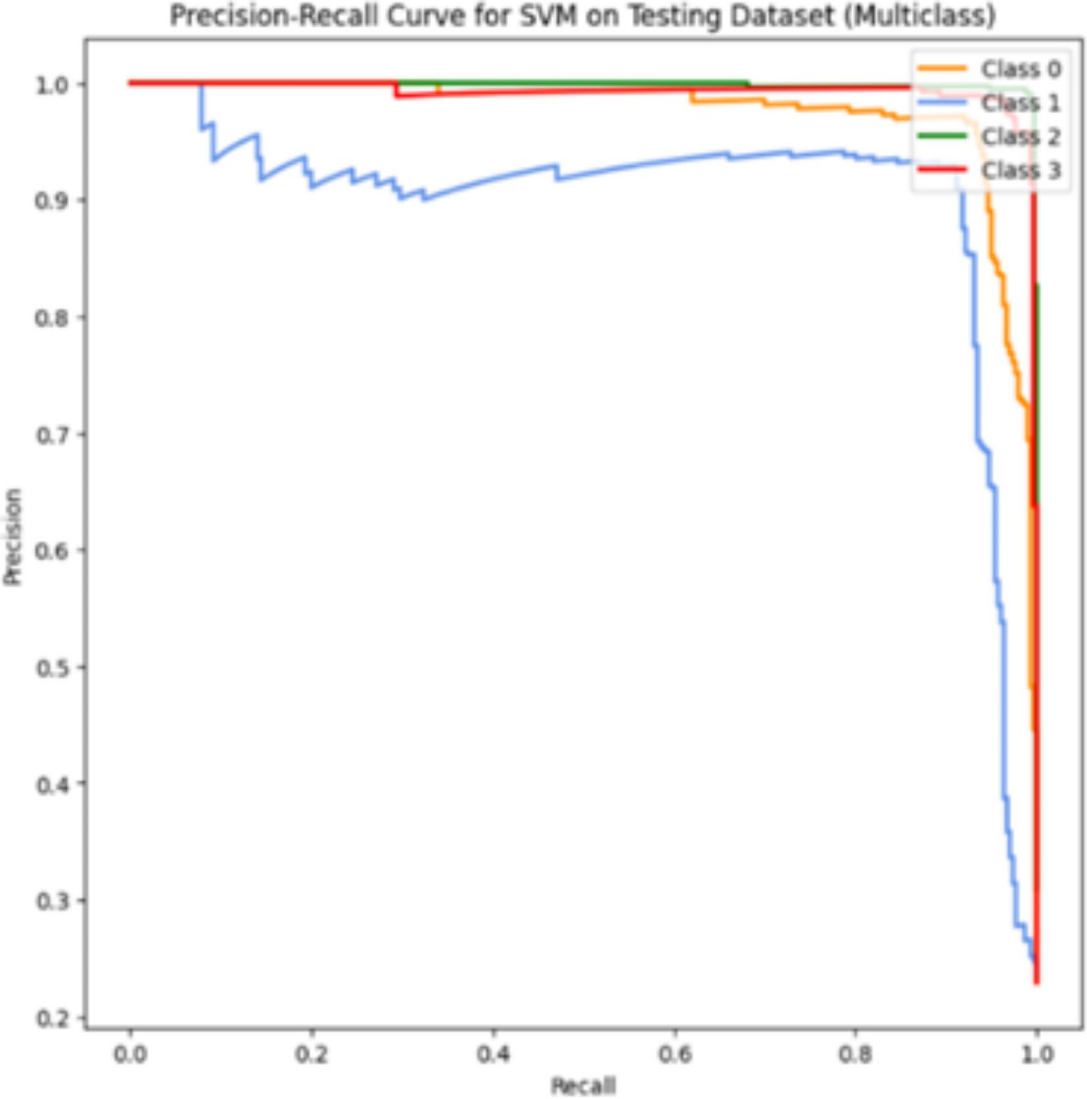
Precision Recall Curve of SVM with HOG, LBP, PCA on Test Dataset.

Figure 7displays Precision-Recall curve of the high accuracy model. This graph helps us understand how well the classifier performs across different thresholds. The curve illustrates the relationship between Precision and Recall. The graph shows how precision and recall change as the classifier’s threshold varies^36^. In our study, we analyze PR curves for four classes: glioma (dark orange, Class 0), meningioma (cornflower blue, Class 1), no tumor (green, Class 2), and pituitary (red, Class 3). The curves draw prec_val on the y-axis and rec_val on the x-axis. The no tumor class (green) achieves the highest performance indicating perfect precision and recall. The pituitary class (red) follows, with precision initially around 0.98 and reaching 1 towards the end, showing strong performance in identifying positive cases. The glioma class (dark orange) also reaches high precision and recall values, closely following the pituitary curve. The meningioma class (cornflower blue) exhibits the lowest performance among the four, though it still demonstrates significant precision and recall. The area beneath the PR curve also termed as PR AUC indicates the overall effectiveness of the classifier for each class, with curves closer to the top-right corner signifying superior performance.

**Fig. 8:**
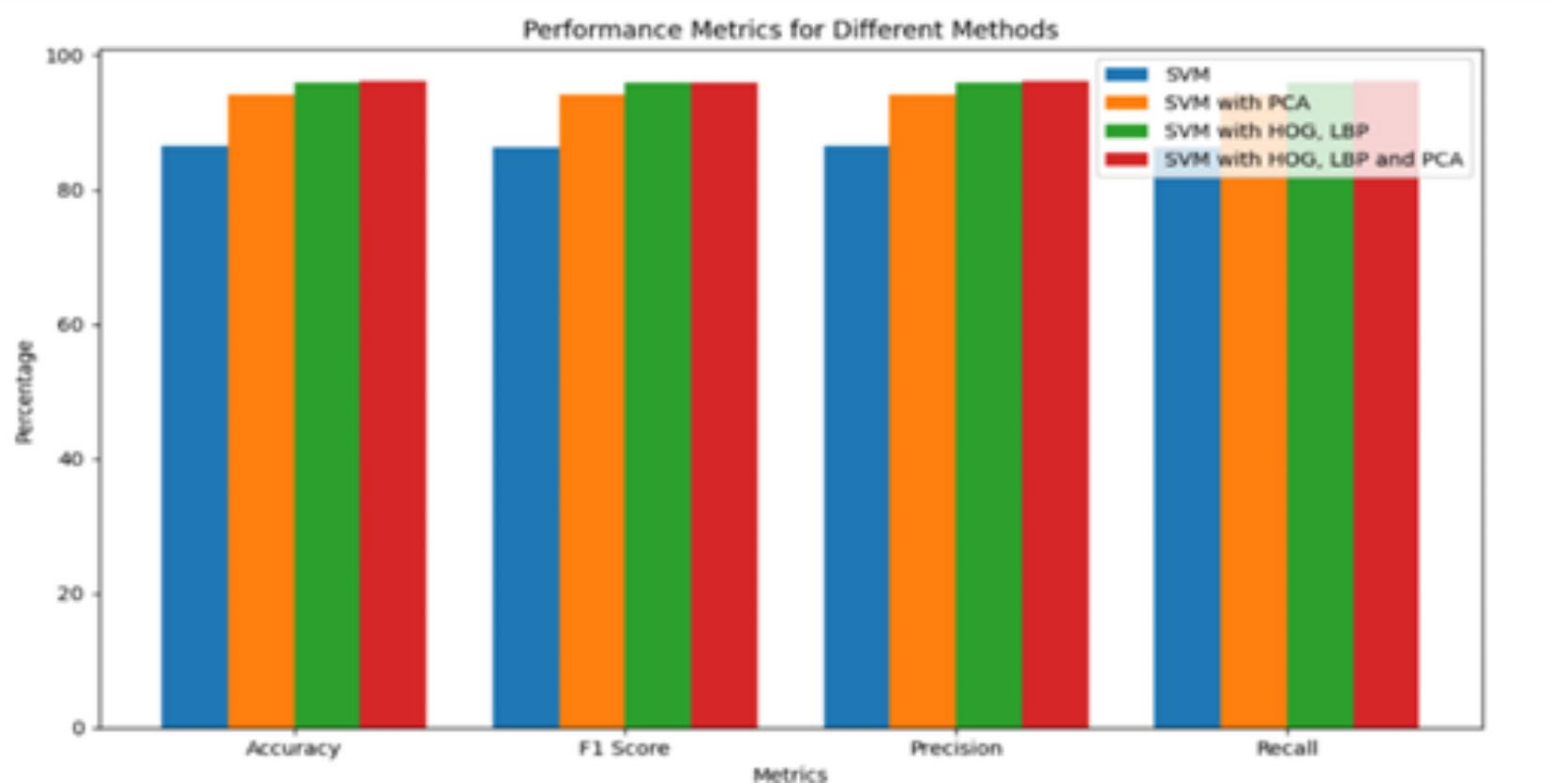
Performance Comparison of SVM Classifiers with Various Feature Combinations.

Figure 8 shows a bar graph comparing the efficacy of SVM classifiers with different combinations of techniques. X-axis displays various metric values such as acc_val, F1_val, prec_val, and rec_val. The y-axis indicates the values of these performance metrics. This graph shows the comparison of SVM classifier’s performance across different feature extraction and dimensionality reduction techniques as described in Table 3.

**Fig. 9:**
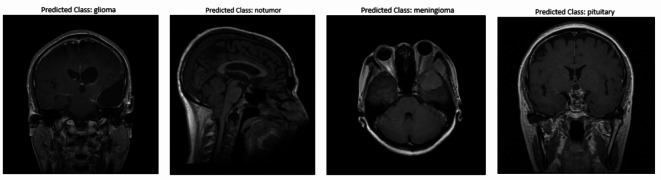
Predictions made by the proposed model.

Figure 9 shows predictions made by the proposed model. The model accurately predicts various brain conditions with a 96% success rate, matching the expected outcomes for each category.

## Discussion

The proposed method is designed to be highly computationally efficient, making it particularly well-suited for medium-sized datasets and resource-limited environments. By integrating advanced feature extraction techniques with SVM, our approach strikes an equilibrium between preciseness and computational efficacy. This method not only competes with but can also surpass more complex, resource-intensive models. This highlights the capability of machine learning techniques to serve as a resource-efficient alternative, offering a viable option where deep learning models may be too demanding in terms of computational resources.

A key distinction of our research lies in its focus on multiclass classification, specifically targeting four different classes of brain tumors, whereas existing studies have predominantly concentrated on binary or ternary classifications. The ability to accurately classify multiple tumor types within a single framework enhances the practical applicability of our method in clinical diagnostics, providing a more comprehensive tool for medical practitioners.

While our methods are optimized for medium-sized datasets, they are also adaptable and scalable to effectively handle larger datasets. As the dataset size increases, so does data’s dimensionality, which can present significant challenges for machine learning models. High dimensionality often leads to increased computational costs and a higher risk of overfitting. To mitigate these challenges, few techniques can be employed. In our study, PCA was used to minimize the dimensional complexity of the data. This reduction not only simplifies the data but also preserves essential information, thereby enhancing model performance and efficiency. Table 4 proves that our proposed method achieved good acc_val, prec_val, rec_val and f1_val as compared to existing studies on the same field.

**Table 4:** Comparison of performance with other related research.

Ref	Year	Method	Number of classes	Acc_val	Prec_val	Rec_val	F1_val
^15^	2020	CNN NADE	3	95%	95%	95%	95%
^16^	2020	Resnet 50	2	95%	-	-	-
^21^	2021	MobilenetV2	2	92%	-	-	-
^27^	2021	VGG 19	3	94%	94%	94%	94%
^28^	2021	CNN SVM	3	95.82%	98%	97.9%	97.9%
^25^	2020	VGG 16	2	95.71%	95%	95.2%	94.7%
^19^	2022	SVM	3	90.27%	90.7%	90.1%	90.2%
^24^	2023	SVM	2	97%	-	-	-
^29^	2022	Multi scale CNN	4	91.2%	90.95%	91.05%	91%
^26^	2023	Xception	4	95.87%	95.84%	95.60%	95.72%
^30^	2023	CNN	4	95.44%	95.32%	95%	95.36%
^31^	2023	2D CNN	4	93.44%	-	-	-
Our developed model: SVM with HOG, LBP and PCA			4	96.03%	96.02%	96.03%	96%

## Conclusion and future work

This research demonstrates the effectiveness of integrating SVM with the methods which are used for extracting the features and reducing the dimensions of data for the categorization of the four types of tumors of the brain using MRI images. By leveraging HOG and LBP for feature extraction and PCA for dimensionality reduction, the study achieved significant enhancement in multiclass categorization accuracy. The baseline accuracy with SVM alone was 86.57%, which increased to 96.03% with the combination of SVM, HOG, LBP, and PCA. The results highlight the potential of these advanced techniques in addressing the challenges of multiclass brain tumor classification and suggest that integrating sophisticated machine learning methods can lead to more accurate and computationally efficient diagnostic tools in medical imaging.

Future work could focus on several key areas to advance the current study. Enhancing feature extraction techniques by exploring methods such as Scale-Invariant Feature Transform could provide new insights into improving model performance. Expanding the dataset to include a greater variety of disease classes and incorporating data from diverse sources could enhance the model’s generalizability and robustness. Validating the model with real-world clinical data would be crucial for assessing its practical applicability. Finally, integrating and evaluating alternative ensemble methods, such as Gradient Boosting Machines (GBM), could further optimize classification accuracy and overall effectiveness.

## Data Availability

The data that support the findings of this study are available from the corresponding author, upon reasonable request.
